# Colorimetric Sensing of the Peroxide Number of Milk Powder Using CsPbBr_3_ Perovskite Nanocrystals

**DOI:** 10.3390/bios13040493

**Published:** 2023-04-20

**Authors:** Li Zhang, Yimeng Zhu, Zhiyong Guo, Longjie You, Chen Zhang, Xi Chen

**Affiliations:** 1 Institute of Analytical Technology and Smart Instruments, College of Environment and Public Healthy, Xiamen Huaxia University, Xiamen 361024, China; zhangl@hxxy.edu.cn (L.Z.);; 2 State Key Laboratory of Marine Environmental Science, Xiamen University, Xiamen 361005, China; zhuym@xmu.edu.cn; 3 National Quality Supervision and Inspection Center for Incense Products, Yongchun 362600, China

**Keywords:** peroxide number, perovskite nanocrystals, CsPbBr_3_, photoluminescence wavelength shift, colorimetric sensing

## Abstract

In this study, a wavelength-shift-based colorimetric sensing approach for the peroxide number of milk powder using CsPbBr_3_ perovskite nanocrystals (CsPbBr_3_ NCs) has been developed. Through the fat extraction, REDOX reactions and halogen exchange, as well as the optimized experimental conditions, a colorimetric sensing method was established to determine the peroxide number of milk powder samples. The integrated process of milk powder fat extraction and the REDOX process greatly shortened the determination time. This colorimetric method has a good linear correlation in the range of the peroxide number from 0.02 to 1.96 mmol/kg, and the detection limit was found to be 3 μmol/kg. This study further deepens the application prospect of wavelength-shift-based colorimetric sensing using CsPbBr_3_ NCs.

## 1. Introduction

Milk powder is a kind of food obtained from fresh animal milk after the moisture removal and drying process. It preserves the high protein and calcium content for dairy usage and has become an important and common human food. Similar to edible oil, milk powder also contains a large amount of fat embedded in the granular structure and a small amount of free fatty acids. Infant milk powders contain more than 20% fat and approximately half of it is unsaturated fatty acids, which makes them more prone to oxidation and deterioration [1,2,3]. In the air, these active unsaturated fatty acids are easily oxidized to peroxides and then degraded into aldehydes and ketones [4], which affects the nutritional value [5] and the taste of the milk powder and reduces the shelf life. As peroxides can destroy the structure of cell membrane, the long-term consumption of foods, including the oxidized milk powder, with excessive peroxides is very harmful to human health [5,6,7] due to the high possibility of cardiovascular diseases, cancer and other chronic diseases. To date, there have emerged several quality testing methods for milk powder [8,9,10,11]. Generally, the oxidation degree of fats or fatty acids could be indicated as a peroxide number, reported as the gram equivalent of iodine in 100 g of a test sample [12], which indicates the quantity of the peroxides in samples. Therefore, the determination of the peroxide number is an important part of the quality control of milk powder to improve quality and prevent potential hazard to human health. At present, the International Standard, ISO 3976:2006 [13], could be known as the most reliable and standardized method for the peroxide number determination of milk powder. In this method, the peroxide in a milk powder sample reacts with ferrous chloride, and the resulting Fe^3+^ yields a red complex with ammonium thiocyanate. According to ISO 3976:2006, spectrophotometry is a widely used method for the determination of the peroxide number of milk powder, but there are still several limitations, such as: (1) the preparation process for the determination is relatively complex and time-consuming; (2) the test solutions need to be prepared fresh; and (3) the sensitivity is still unsatisfactory. Recently, several approaches for the analysis of edible oil oxidation or type based on electrical conductivity [14] and surface-enhanced Raman spectroscopy (SERS) [15,16,17] have been reported, which provide new routes for the rapid determination of the peroxide number using a device. In addition, simple and rapid colorimetric sensing approaches could also be considered.

**Figure 1:**
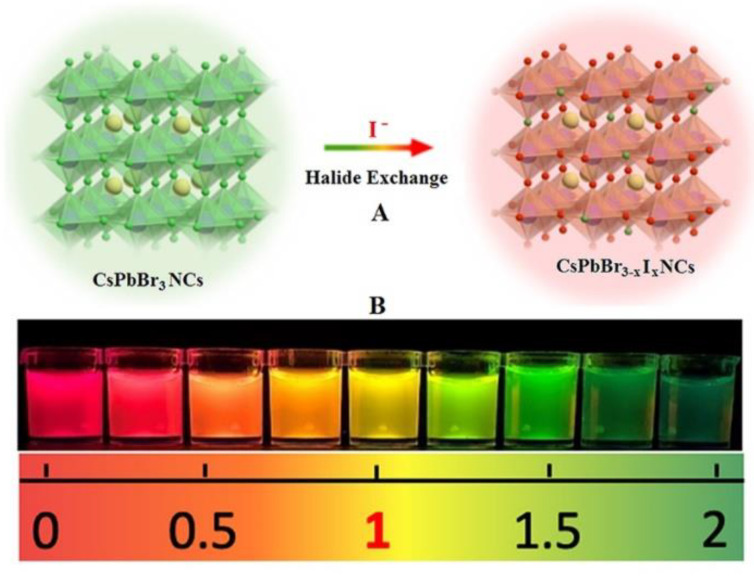
Halogen exchange of CsPbBr_3_ NCs with I^−^ (**A**), and a standard colorimetric card for different peroxide numbers (**B**).

Generally, colorimetric sensing provides more convenient and sensitive approaches compared with those based on the luminescence intensity change because the human eye exhibits higher sensitivity to color changes. Recently, colorimetric sensing studies and applications of CsPbBr_3_ perovskite nanocrystals (CsPbBr_3_ NCs) have drawn researcher attention [18]. CsPbBr_3_ PNCs exhibit excellent luminescence properties, such as high quantum yield, narrow full width at half maximum (FWHM) [19,20] and ion exchange [21]. They have been widely used in solar panels [22], light-emitting diodes [23,24], laser fields [25], UV blocking [26], and have revealed excellent application prospects in the field of sensing [18,27,28,29]. The change in the proportion of halogen ions in the CsPbBr_3_ NCs structure will cause the change in their fluorescence emission wavelength, as shown in Figure 1A, which will cover the whole visible spectrum region and produce a significant color change from blue to red. The above phenomena are very beneficial for the establishment of wavelength-shift-based colorimetric sensing without any additional materials. Recently, several colorimetric sensing studies have been reported using the wavelength-shift of the halogen exchange characteristics of C_S_PbX_3_ NCs, in which the concentrations of Cl^−^ and methylamine (MA) gas, as well as the peroxide number of edible oil, have been detected [18,29,30,31]. In addition, based on the similar consideration, colorimetric sensing approaches have been developed for the determination of chlorine, iodine and other trace elements in tap water [32], H_2_S in rat brain microdialysate [27] and benzoyl peroxide in flour and noodle samples [33].

The development of rapid colorimetric sensing approaches for the peroxide number of milk powder will greatly improve the detection efficiency and facilitate the quality control of milk powder. In this study, a colorimetric sensing method using CsPbBr_3_ NCs was developed and applied to the rapid colorimetric sensing of the milk powder peroxide number. Considering the fat substances need to be extracted from the milk powder for the subsequent process, we established the three processes, including the milk powder fat extraction, REDOX reaction and halogen exchange. As both the process of fat extraction and the REDOX process between oleylammonium iodide (OLAM-I) and peroxides in milk powder can occur in the same organic solvent (*n*-hexane/isopropanol), the REDOX reaction and the fat extraction from milk powder can be realized at the same time for less pre-preparation time and a simpler determination process. The experimental results reveal that this approach is applicable for the rapid visual determination of the peroxide number of milk powder samples.

## 2. Materials and Methods

### 2.1. Materials and Chemicals

Oleic acid (OA, 90%), octadecene (ODE, 90%), oleylamine (OAM, 80–90%), CS_2_CO_3_ (99.9%) and PbBr_2_ (99.99%) were all purchased from Aladin Reagent Co., Ltd. (Shanghai, China). FeCl_3_, MgSO_4_, NaCl, Na_2_S, CaCl_2_, NaI, KCl, NaNO_3_, NaNO_2_, NaClO, Na_3_PO_4_•12H_2_O, NaH_2_PO_4_, Na_2_CO_3_, NaHCO_3_ and Zn(NO_3_)_2_•6H_2_O were purchased from Sinoptic Chemical Reagents Co., Ltd. (Shanghai, China). All other reagents were at least analytical grade and without further purification. The water was used throughout the experiments using the water purification system (Millipore, Burlington, MA, USA).

### 2.2. Instruments

The absorption spectra were characterized by a Hitachi UV-Vis 2550 spectrophotometer. Fluorescent spectra of CsPbBr_3_ NCs were collected by an F-4500 fluorescence spectrophotometer (Hitachi, Tokyo, Japan). The in-situ halogen exchange between Br^−^ and I^−^ was observed using a Renishaw Invita Raman (Renishaw, Wotton-under-Edge, UK) spectrometer with a laser excitation source (457 nm). The morphology of CsPbBr_3_ NCs was characterized by JEOL-1400 transmission electron microscopy (TEM, Tokyo, Japan), and the relevant acceleration voltage was set at 100 kV. A UV-2550 spectrophotometer (Shimadzu, Shenzhen, Japan) was used for the determination of the peroxide number of milk powder following the ISO international method. In the synthesis of CsPbBr_3_ PNCs, a magnetic stirrer with electric heating sleeve (ZNCL-TS, Shanghai, China) and a high-speed centrifuge (TG16-WS, Hunan, China) were employed.

### 2.3. Preparation of CsPbBr_3_ NCs and 9-Octadecenyl Iodide Amine

CsPbBr_3_ NCs was prepared according to the reference [34], but the dosage was modified in order to obtain the larger amount of products. In the first step, cesium oleate precursor was prepared by adding 0.1628 g of Cs_2_CO_3_, 8 mL of octadecene and 0.5 mL of oleic acid into a three-necked flask. The reactants were heated and stirred until Cs_2_CO_3_ was completely dissolved at 120 °C under a vacuum condition (about 40 min). Nitrogen was aerated into the flask and kept stirring at 150 °C under the nitrogen atmosphere. In the second step, CsPbBr_3_ NCs could be obtained by the thermal injection method [26]. In the synthesis, 0.138 g of PbBr_2_, 10 mL of octadecene, 1 mL of oleic amine and 1 mL of oleic acid were put into a three-necked flask. The reactants in the flask were heated and stirred until PbBr_2_ was completely dissolved at 120 °C under a vacuum condition (approximately 30 min), and then kept stirring at 150 °C in nitrogen atmosphere. Next, 0.8 mL of cesium oleate precursor solution was quickly injected into the stirred solution, the reaction mixture was quickly cooled down using an ice water bath and then returned to room temperature. The obtained product, CsPbBr_3_ NCs, was finally purified by high-speed centrifugal separation at 10,000 rpm for 10 min. The product was re-dispersed in *n*-hexane solution, and 0.5 mg/mL CsPbBr_3_ NCs was used for further experiments.

Finally, 9-octadecenyl iodide amine (OLAM-I) was synthesized by stirring iodine and oleamine using the high temperature method as in the report [35]. The product was naturally cooled and then diluted by adding a mixture of *n*-hexane and isopropanol (3:1, *v*/*v*) to obtain the final OLAM-I diluent.

### 2.4. Preparation of Standard Colorimetric Card

In order to guarantee the representative peroxide number for different samples, ten newly purchased milk powder samples of different brands in local supermarkets were collected and mixed. In the preparation of comparative standard milk powder samples, about 10 g of the mixed milk powder sample was placed in a watch glass with a thickness of about 1 cm for the full oxidation with the oxygen in the air. The peroxide number of this milk powder (about 2 mmol/kg) was accurately determined according to ISO 3976:2006. In the preparation of the standard colorimetric card, 0.3 g of the above comparative standard milk powder was mixed with 10 mL of OLAM-I solution, stirred at 1500 rpm in a sealed bottle for 10 min, and then placed for 2 min. The supernatant with a volume of 0 mL (blank), 0.1 mL, 0.2 mL, 0.25 mL, 0.3 mL, 0.35 mL, 0.4 mL, 0.45 mL and 0.5 mL was added into a centrifuge tube, respectively, and then OLAM-I solution was added the centrifuge tube until the total volume was 0.5 mL. A series of standard extracts with different peroxide numbers were prepared, and the peroxides reacted with the excess OLAM-I. The halogen exchange occurred between the excess OLAM-I and 0.5 mg/mL CsPbBr_3_ NCs to yield CsPbBr_3−x_I_x_NCs which emits at a different photoluminescence wavelength. As shown in Figure 1B, after the reaction with OLAM-I and CsPbBr_3_ NCs, the above prepared supernatant with different peroxide numbers revealed obviously different colors. The corresponding peroxide number for the supernatant could be quantitatively determined using ISO 3976:2006. A standard colorimetric card could be prepared by the present color and the peroxide number obtained by the ISO method. The milk powder sample quality is satisfactory if its peroxide number is lower than 0.5 mmol/kg, while serious oxidation has occurred and it is not suitable for further consumption if the peroxide number is higher than 1 mmol/kg [36]. Therefore, a point line at 1 mmol/kg is highlighted in the standard colorimetric card.

### 2.5. Colorimetric Sensing for the Peroxide Number of a Milk Powder Sample

In the determination or sensing of the peroxide number, the fat extraction in milk powder is an important procedure, which is different from such samples as edible oils [29]. As the fatty substances in milk powder are embedded in the matrix, a suitable solvent will increase the extraction efficiency. Pearce, D. et al. extracted the fatty substances from milk powder using *n*-hexane and anhydrous sodium sulfate [37]. Stefania C. et al. used *n*-hexane/isopropanol as the extraction solvent for the extraction, and the extraction rate was over 80%, which greatly improved the determination accuracy of the peroxide number of milk powder [38].

**Figure 2:**
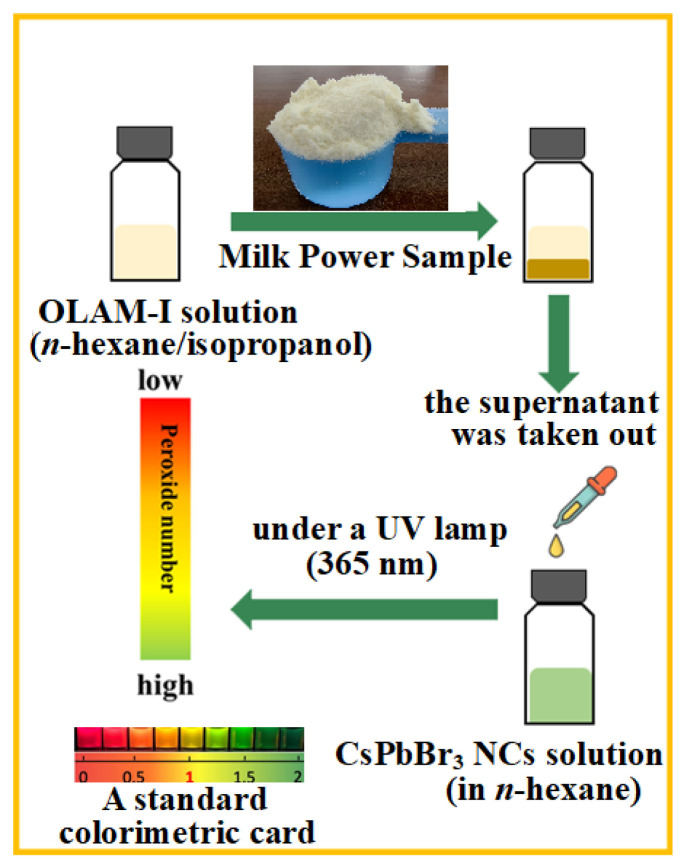
General procedure for the colorimetric sensing of the peroxide number of milk powder.

In the experiments, the process of fat extraction is accompanied by the REDOX reaction. As shown in Figure 2, a certain amount of the milk powder sample was firstly added into OLAM-I *n*-hexane/isopropanol solution, and the REDOX reaction between OLAM-I and the peroxides in the milk powder sample occurred at the same time in the extraction. The supernatant was then extracted and added to CsPbBr_3_ NCs for the halogen exchange. Finally, according to the apparent color of CsPbBr_3−x_I_x_NCs, the peroxide number of the milk powder sample could be indirectly identified using the colorimetric card. The sensing procedure generally included: (1) about 0.3 g of milk powder sample was added into 0.5 mg/mL OLAM-I solution (*n*-hexane/isopropanol, *v/v* 3:1, 10 mL) in a sealed reaction vessel. The suspension was stirred 12 min at a rate of 1500 rpm. The supernatant could be obtained after the suspension was placed for 2 min; then, (2) 0.5 mL of the supernatant was added to 0.5 mg/mL CsPbBr_3_ NCs solution (in 1 mL *n*-hexane) for 5 min rapid halogen exchange; and (3) the presented color of the solution under a UV lamp (365 nm) was compared with the colorimetric card to estimate the peroxide number of the milk powder sample. Using the colorimetric card, its quality could be identified according to the color range exceeding the qualified line or not.

As a comparison method, the international standard (ISO 3976:2006) was used for the determination of the peroxide number of the milk powder sample and the detail procedures are shown in the support information.

## 3. Results and Discussion

### 3.1. Solvent Selection for the Fat Extraction

The peroxide number of edible oils could be determined by the halogen exchange of CsPbBr_3_ NCs with the I^−^ anions from the residual OLAM-I after the REDOX reaction between OLAM-I and peroxides [29]. After the exchange with CsPbBr_3_ NCs, the fluorescence emission wavelength of the product, CsPbBr_x_I_3−x_ NCs, will shift greatly and result in the obvious change of the emission color, which is conducive to colorimetric sensing. Similar to edible oils, after extracted, the fat in milk powder can participate in the REDOX reaction with OLAM-I to affect the degree of halogen exchange with CsPbBr_3_ NCs. In the colorimetric sensing for milk powder, the fat extraction process can simultaneously make the REDOX reaction with OLAM-I in the same organic solvent, which shortens and simplifies the sensing process. According to the report [37], as shown in Table S1, the fat extraction rate using *n*-hexane/isopropanol (3:1, *v*/*v*) can reach more than 80%, which is the highest rate among the commonly used extraction solvents in the fat extraction. In addition, the polarity of isopropanol should be taken into account because the polar solvent will cause damage to the CsPbBr_3_ NCs structure. In order to ensure the stability of CsPbBr_3_ NCs in *n*-hexane/isopropanol (3:1, *v*/*v*), the PL emission spectrum of CsPbBr_3_ NCs was observed as shown in Figure S2 (the green line), indicating the maximal emission wavelength is at 520 nm, which is the same as that in toluene [29]. No obvious PL intensity change could be observed, even if CsPbBr_3_ NCs was dissolved and stored in *n*-hexane/isopropanol for more 6 h. Furthermore, OLAM-I could be completely dissolved after shaking. The halogen exchange reaction between OLAM-I and CsPbBr_3_ NCs could smoothly occur in *n*-hexane/isopropanol (3:1, *v*/*v*), and the red shift of the PL emission wavelength as shown Figure S2 (the yellow line) could be observed. In this experiment, *n*-hexane/isopropanol (3:1, *v*/*v*) could be selected for the milk powder fat extraction and as a solvent to dissolve OLAM-I and CsPbBr_3_ NCs for the halogen exchange reaction.

The process of extracting fat from milk powder was accompanied by REDOX reactions between the peroxides in the fat and OLAM-I. With the addition of *n*-hexane/isopropanol solvent, the fat was released slowly and participated in the REDOX reactions between peroxides in the fat and OLAM-I, and gradually the solution was observed to turn slightly yellow. Generally, a longer extraction time would be helpful to ensure the degree of extraction completion, but unsuitable for high analytical efficiency. Obviously, with the extraction time increase, as shown in Figure S3 (Supplementary Materials), the maximal PL wavelength gradually blue-shifted because the REDOX reactions were approaching completion, and more iodide ions from OLAM-I were consumed, which reduced the halogen exchange with CsPbBr_3_ NCs to CsPbBr_3−x_I_x_ NCs. When the extraction time was longer than 12 min, the maximal PL wavelength tended to be stable.

In order to study the effect of temperature on the extraction of fat and the reaction between peroxides in the fat and OLAM-I, the PL spectra of CsPbBr_3_ NCs at different extraction temperatures (25 °C, 30 °C, 40 °C, 50 °C and 60 °C) were measured. As shown in Figure S4, the maximal PL wavelength of CsPbBr_3_ PL revealed no significant shift at the different extraction temperature. This result indicates that the extraction temperature in the range of 25 °C to 60 °C has little effect on the sensing result, and the REDOX reactions have completed synchronously. In order to facilitate the practical application, room temperature (about 25 °C) was used as the extraction temperature to further study.

In the fat extraction, a suitable stirring rate would be helpful to release the fat in milk powder. The lower stirring rate caused longer extraction time, but a too high stirring rate would lead to the protein flocculation, which is not conducive to the extraction of fat. In the experiment, the stirring rate of 1500 rpm was selected.

Following the above results, in the sensing of the peroxide number of a milk powder sample, the powder sample was extracted using *n*-hexane/isopropyl alcohol (3:1, *v*/*v*) containing 0.5 mg mL^−1^ OLAM-I for 12 min at room temperature at a stirring rate of 1500 rpm. After the extraction and the REDOX reactions, the suspension was placed for 2 min and then 0.5 mL of the supernatant from the placed suspension was added to 0.5 mg mL^−1^ CsPbBr_3_ NCs solution (in 1 mL *n*-hexane,) for 5 min halogen exchange reaction.

### 3.2. Performance Evaluation for the Colorimetric Sensing of the Peroxide Number Using CsPbBr_3_ NCs

**Figure 3:**
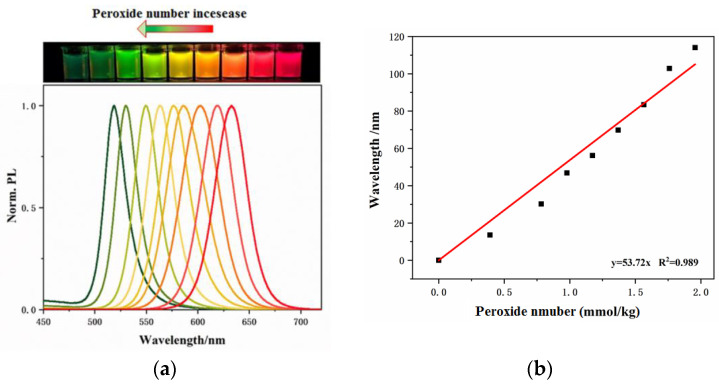
(**a**) The blue shift of the PL wavelength of CsPbBr_3_ NC with the increase of the peroxide number, and (**b**) linear relationship between the wavelength shift and the peroxide number.

A colorimetric sensing method using CsPbBr_3_ NCs was established to detect the peroxide number of a milk powder sample based on the above optimized conditions. In the experiment, a series of extract standard samples with different peroxide numbers was prepared following the procedures as described in Section 2.4. The corresponding peroxide numbers of the above standard samples were determined according to ISO 3976:2006. Using the standard samples, the corresponding PL wavelength shift for their different peroxide numbers could be obtained by CsPbBr_3_ NCs colorimetric sensing method. As shown in Figure 3, the blue shift of the PL wavelength of CsPbBr_3_ NCs coincides with the peroxide number increase in the standard sample because the higher content of peroxides (larger peroxide number) consumes more I^−^ from OLAM-I, which reduces the halogen exchange with CsPbBr_3_ NCs. Correspondingly, the color presented from red, orange, yellow, green yellow, and finally to light green as shown in Figure 3a (the up figure). The relationship between the quantitative peroxide number of the standard samples and their corresponding maximal PL wavelength is listed in Table S2. The maximal PL wavelength was found to be 633 nm when the peroxide number was 0. With the increase of the peroxide number to 1.96 mmol/kg, the maximal PL wavelength shifted to 519 nm. As shown in Figure 3b, the peroxide number of the standard sample presented a good linear relationship in the range from 0.02 mmol/kg to 1.96 mmol/kg with the PL wavelength shift, and the linear regression equation of y = 53.72x (y, wavelength shift, x, peroxide number) with a correlation coefficient (R^2^) of 0.989 could be obtained. The limit of detection (LOD) of the peroxide number is 3 μmol kg^−1^ (LOD = 3σ/k, k = 53.72, the slope of linear equation, σ is relative standard deviation).

### 3.3. Reproducibility of the Colorimetric Sensing Approach

In order to investigate the reproducibility of the sensing method, the multiple sensing tests for a same batch of milk powder sample were carried out. After six parallel samplings and extractions for the samples, each of the 0.5 mL supernatants was added to CsPbBr_3_ NCs solution, respectively. After the halogen exchange, their PL spectra and presented colors were observed and recorded. In addition, one of the above six supernatant samples was selected and tested six times to investigate the reproducibility of the same extraction. As shown in Table S3, the relative standard deviation (RSD) of the maximal PL wavelength for the same batch of milk powder sample was found to be 0.16%, and the RSD of the maximal PL wavelength for the six supernatant samples was 0.11%. The RSD values are all less 0.2%.

### 3.4. Colorimetric Sensing of the Peroxide Number for Milk Powder Samples Using CsPbBr_3_ NCs

Based on the above optimal experimental conditions, the colorimetric sensing method of the peroxide number for milk powder samples using CsPbBr_3_ NCs was established. As shown in Figure S5 and Video S1, the whole detection process took about 20 min, including the fat extraction and REDOX reaction, halogen exchange and colorimetric process. The peroxide number of milk powder samples could be tested by comparing with the final sensing color and the standard colorimetric card. As listed in Table 1, several milk powder samples were newly purchased with sealed packaging from local markets. Sample 1 was a defatted milk powder and sample 2 to sample 4 were whole milk powder samples. Sample 5 was prepared by exposing the whole milk powder sample (sample 2) to air for two weeks. Comparing with the standard colorimetric card as shown in Figure 1, the red color of the test solution indicates the low peroxide number and edibility, but the light yellow color, as in sample 5, indicates the critical level of peroxides for safe consumption. As listed in Table 1, the colorimetric sensing color was bright red for sample 1, red for sample 2, pink for sample 3, reddish orange for sample 4 and dark yellow for sample 5. The sensing results show that the peroxide number is very low for the defatted milk powder sample (sample 1). Although the peroxide number for samples 2, 3 and 4 is obvious larger than that of sample 1, the values are all in the safe range (lower 0.5 mmol/kg). The peroxide number of sample 2 increased from 0.2 mmol/kg to 1.1 mmol/kg after it was exposed to air for two weeks, indicating the obvious influence of oxygen. The sensing results are consistent with those from ISO 3976:2006, by which the peroxide number of sample was found to be 0.05, 0.15, 0.32 and 0.45 mmol/kg for sample 1 to sample 4, respectively, and the highest peroxide number of 1.04 mmol/kg of sample 5 was obtained.

## 4. Conclusions

In this study, a colorimetric sensing method using CsPbBr_3_ NCs was developed and successfully applied to the rapid determination of the peroxide number for milk powder. The synchronized process of the fat extraction and the REDOX reactions between the peroxides and OLAM-I simplified the procedure and reduced the sensing time. The entire procedure could be finished in 20 min. The PL color could be directly compared with the standard colorimetric card to obtain the peroxide number of the milk powder samples, by which the peroxides in the sample could be evaluated. In addition, the quantitative analysis for the peroxide number of the milk powder samples could be realized based on the PL wavelength shift, and the experimental results revealed that the wavelength shift presented a good linear correlation in the range of the peroxide number from 0.02 to 1.956 mmol/kg, and the detection limit was found to be 3 μmol/kg.

## Figures and Tables

**Table 1:** Determination of the peroxide number for milk powder.

	Sample 1	Sample 2	Sample 3	Sample 4	Sample 5
Sensing color	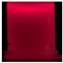	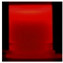	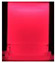	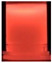	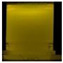
Sensing results (mmol/kg)	0.1	0.2	0.3	0.4	1.1
ISO test result (mmol/kg)	0.05	0.15	0.32	0.45	1.04
Quality	Edible	Edible	Edible	Edible	Inedible

Sample 1, new defatted milk powder; Sample 2, 3 and 4, different brand of new whole milk powder samples; Sample 5, the sample 2 was stored in air for 2 weeks.

## Data Availability

The data are available under request to the correspondence.

## Supplementary Materials

The following supporting information can be downloaded at: https://www.mdpi.com/article/10.3390/bios13040493/s1, Figure S1: Calibration curve of spectrophotography; Figure S2: The fluorescence emission spectra of CsPbBr3 NCs (green line) and red shift of induced by OLAM-I (orange-yellow line) in n-hexane/isopropanol (*v*/*v*); Figure S3: Maximal PL wavelength at different extraction time; Firure S4: Maxmal PL emission wavelength of CsPbBr3 NCs after the halogen exchange with OLAM-I at the different extraction temperature; Figure S5: Photo records of the colorimetric sensing for peroxide number of powder milk using CsPbBr3 NCs; Table S1: Extraction rate of fat using different organic solvent [15,16,17]; Table S2: Peroxide number, wavelength and wavelength shift of the standard samples; Table S3: Reproducibility of the colorimetric sensing method; Video S1: Video record of the colorimetric sensing for peroxide number of powder milk using CsPbBr3 NCs.
