# Supplementary material for: Colorimetric Sensing of the Peroxide Number of Milk Powder Using CsPbBr3 Perovskite Nanocrystals
Source: Biosensors (Basel). 2023 Apr 20;13(4):493. doi: 10.3390/bios13040493 (PMC10137039; doi:10.3390/bios13040493)
Supplement: Supplementary file 1 [file biosensors-13-00493-s001.zip › biosensors-2318617-supplementary.pdf]

Support Information:

Colorimetric Sensing of the Peroxide Number of Milk Powder Using $CsPbBr_3$ Perovskite Nanocrystals

Li Zhang [1,†], Yimeng Zhu [2,†], Zhiyong Guo [1], Longjie You [3], Chen Zhang [1] and Xi Chen [2,*]

1 Institute of Analytical Technology and Smart Instruments, College of Environment and Public Healthy, Xiamen Huaxia University, Xiamen 361024, China; zhangl@hxxy.edu.cn (L.Z.)
2 State Key Laboratory of Marine Environmental Science, Xiamen University, Xiamen 361005, China; zhuym@xmu.edu.cn
3 National Quality Supervision and Inspection Center for Incense Products, Yongchun 362600, China
* Correspondence: xichen@xmu.edu.cn
† These authors contributed equally to this work.

For the international standard (ISO 3976:2006, "Milk Fat - Determination of the peroxide number"), the principle is: the reaction of the peroxides in the milk powder sample and ferrous chloride generates Fe^{3+}, and a red complex will be produced after the reaction of Fe^{3+} and ammonium thiocyanate. The content of the peroxides can be calculated by iron complex absorbance. The specific steps are as follows: (1) Reagent blank: The blank solution contains 9.90 mL of methanol/1-decanol/*n*-hexane (3:2:1, v/v/v) mixture, 0.05 mL of ammonium thiocyanate aqueous solution (30 g/100 mL) and 0.05 mL of iron chloride solution ($C_{Fe}{}^{2+}$ = 1 mg/mL). The mixture is transferred to a cuvette. After being placed for 10 minutes, the absorbance of the mixture is taken as the reagent blank, E_1. (2) Sample blank: 0.33g of milk powder sample (Mo) is mixed with 9.60 mL methanol/1-decanol/*n*-hexane (3:2:1, v/v/v) in a tube by gently mixing to dissolve the fat of the sample. After being filtered, the supernatant is mixed with 0.05 mL ammonium thiocyanate. The solution is then transferred to a cuvette and placed for 10 minutes. The absorbance of the solution is taken as the sample blank (E_O'). Using the formula Eo= E_O' X (M/Mo), the corrected absorbance blank of the sample can be obtained, where: Eo is the absorbance value of the corrected blank of the sample; E_O' is the absorbance blank value of the sample; Mo is the amount for the sample blank test; and M is the amount of the test sample. (3) Sample test: 0.33g of milk powder sample is weighed (M) and then added to 9.60 mL methanol/1-decanol/*n*-hexane mixture. After being gently mixed to dissolve the fat in the sample, 0.05 mL of ammonium thiocyanate

solution and 0.05 mL of ferrous chloride solution are added and mixed again. The mixture is transferred to a cuvette, and placed for 10 minutes. The absorbance (E_2) is finally measured. (4) Absorbance coefficient: 0.5 mL, 1.0 mL, 1.5 mL, 2 mL, 3 mL, 4 mL and 5 mL ferric chloride standard solution ($C_{Fe^{3+}}$ = 0.01 mg/mL) are added to different test tubes, respectively, to obtain a series containing 5 μg, 10 μg,15 μg, 20 μg, 30 μg, 40 μg and 50 μg of Fe^{3+} solution. Different volumes of methanol/1-decanol/*n*-hexane are added to ensure the mixture volume of 9.9 mL in each tube. Then 0.05 mL of ammonium thiocyanate solution and 0.05 mL of hydrochloric acid solution are added and mixed. The obtained reaction mixture is transferred to a cuvette. They are closed and allowed to stand for 10 min. The absorbance (EFE) of methanol/1-decanol/*n*-hexane mixture in the standard solution is tested. As shown in following figure, the linear regression equation Y = 0.0030+ 0.0260X can be obtained, where Y is the EFE value; X is the amount of Fe^{3+}; 0.0030 is the intercept value; and 0.0260 is the value of absorptivity (regression) coefficient. (5) Calculation results: Substituting the above results into the formula, we can get: absorbance E of iron complex = E_2-(E_0+E_1). The amount of Fe^{3+} is Mc=E/ b; The peroxide number PM = 0.5 Mc/55.84 M, and the unit of peroxide number in the test is unified as mmol/kg.

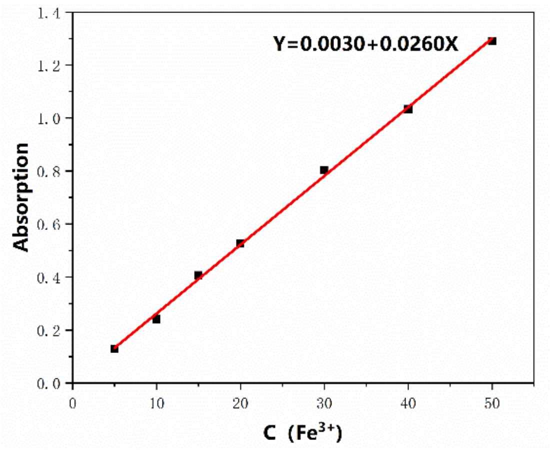

Figure S1. Calibration curve of spectrophotography

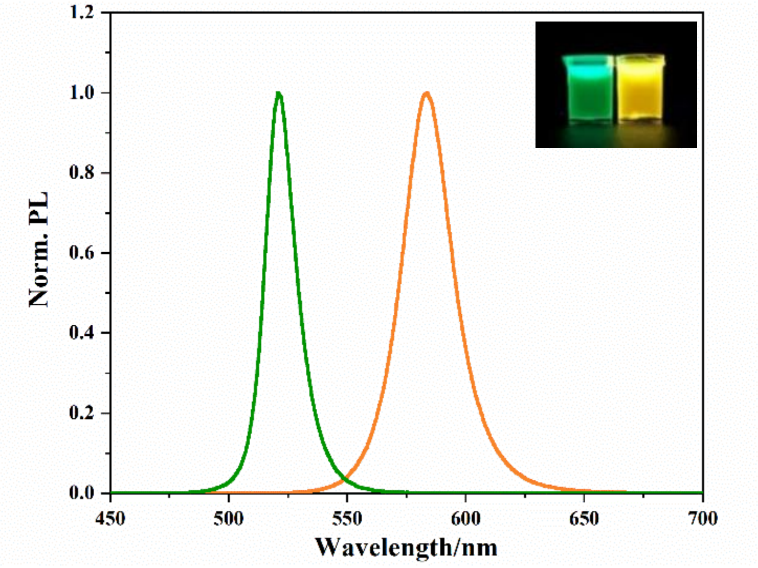

Figure S2. The fluorescent emission spectra of $CsPbBr_3$ NCs (green line) and red shift of induced by OLAM-I (orange-yellow line) in *n*-hexane/isopropanol (v/v)

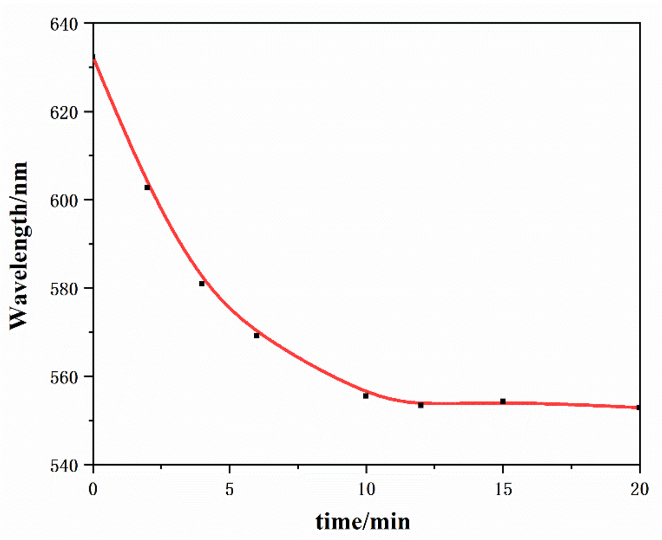

Figure S3. Maximal PL wavelength at different extraction times

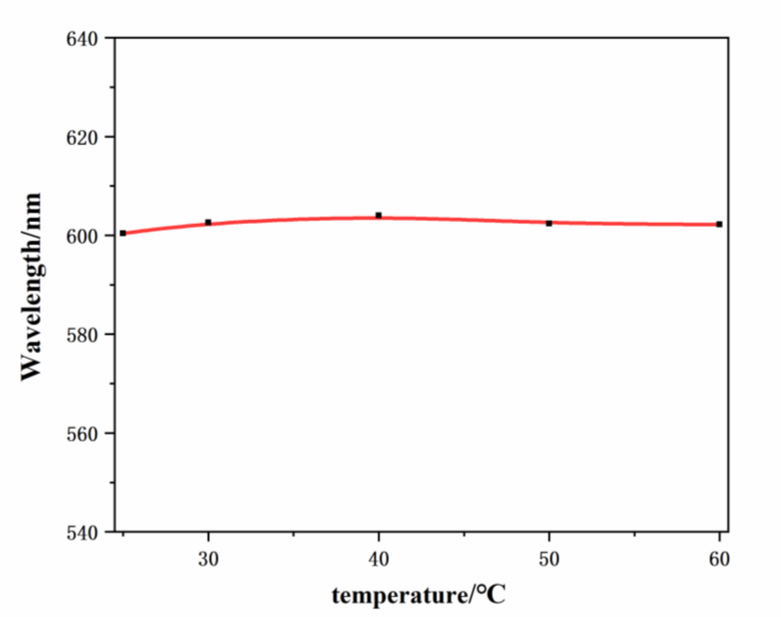

Figure S4. Maxmal PL emission wavelength of $CsPbBr_3$ NCs after the halogen exchange with OLAM-I at different extraction temperatures

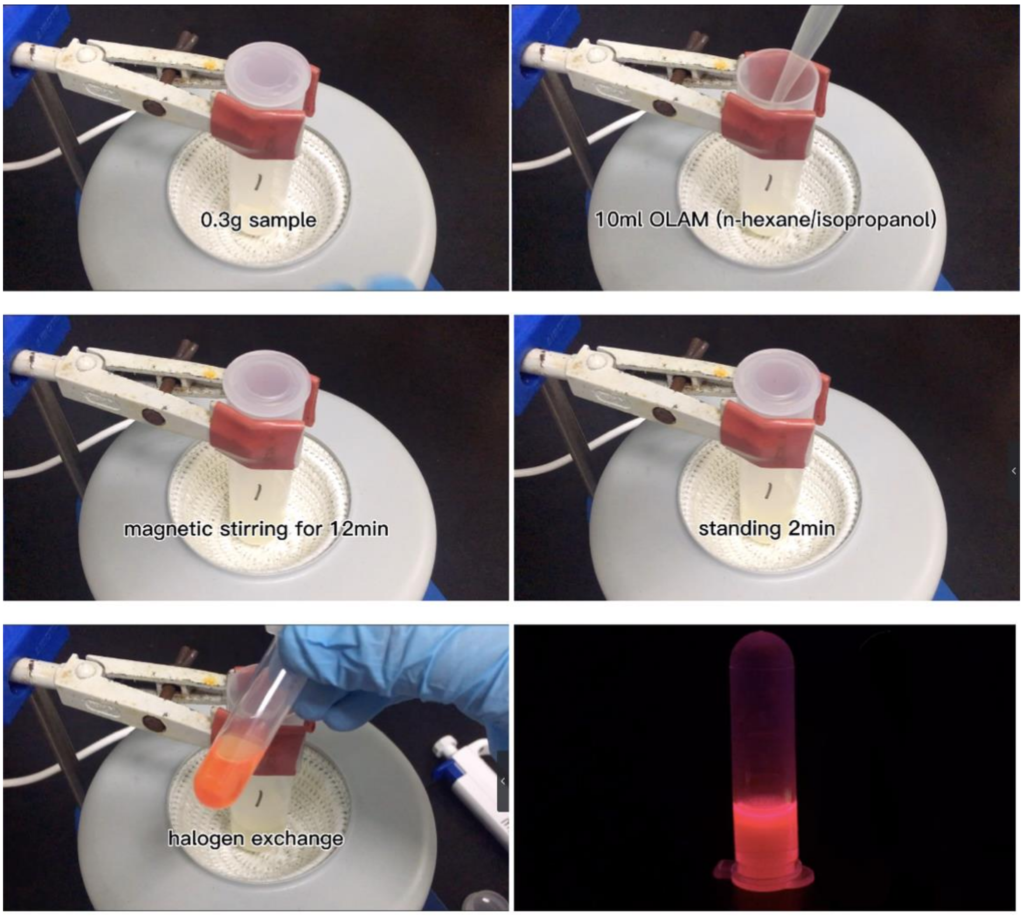

Figure S5. Photo records of the colorimetric sensing for the peroxide number of powder milk using $CsPbBr_3$ NCs

Table S1 Extraction rate of fat using different organic solvents

n-hexane [15]	mortar grinding -*n*-hexane [16]	*n*-hexane /methanol	dichloromethane/methanol [15]	*n*-hexane/ isopropanol [17]
<10%	10% - 60%	<80%	<80%	>80%

Table S2 Peroxide number, wavelength and wavelength shift of the standard samples

Standard samples	1	2	3	4	5	6	7	8	9
Peroxide number* (mmol/kg)	0	0.39	0.78	0.98	1.17	1.37	1.57	1.76	1.96
wavelength (nm)	632.8	619.2	602.6	586.0	576.6	563.0	549.4	530.0	518.8
wavelength shift (nm)	0	13.6	30.2	46.8	56.2	69.8	83.4	102.8	114.0

*Peroxide numbers were detected using ISO 3976:2006.

Table S3 Reproducibility of the colorimetric sensing method

Approach	Sample No.	1	2	3	4	5	6
Parallel extraction for the same milk powder sample	**wavelength (nm)**	622.2	623.0	624.8	624.2	623	624.8
	**RSD**	0.16%					
Parallel sensing of the same extract	**wavelength (nm)**	623	624.8	624.2	623.6	623.2	623
	**RSD**	0.11%					

Reference

15. Jiang, H.; He, Y.C.; Xu, W.D.; Chen, Q.S. Quantitative Detection of Acid Value During Edible Oil Storage by Raman Spectroscopy: Comparison of the Optimization Effects of BOSS and VCPA Algorithms on the Characteristic Raman Spectra of Edible Oils. *Food Anal. Methods* **2021**, *14*, 1826–1835. https://doi.org/10.1007/s12161-020-01939-5.
16. Jiang, Y.; Su, M.; Yu, T.; Du, S.; Liao, L.; Wang, H.; Wu, Y.; Liu, H. Quantitative determination of peroxide value of edible oil by algorithm-assisted liquid interfacial surface enhanced Raman spectroscopy. *Food Chem.* **2021**, *344*, 128709. https://doi.org/10.1016/j.foodchem.2020.128709.
17. Du, S.; Su, M.; Jiang, Y.; Yu, F.; Xu, Y.; Lou, X.; Yu, T.; Liu, H. Direct Discrimination of Edible Oil Type, Oxidation, and Adulteration by Liquid Interfacial Surface-Enhanced Raman Spectroscopy. *ACS Sens.* **2019**, *4*, 1798−1805. https://doi.org/10.1021/acssensors.9b00354.
